# The role of digital health in respiratory diseases management: a narrative review of recent literature

**DOI:** 10.3389/fmed.2025.1361667

**Published:** 2025-02-26

**Authors:** Malik A. Althobiani, Anne-Marie Russell, Joseph Jacob, Yatharth Ranjan, Rami Ahmad, Amos A. Folarin, John R. Hurst, Joanna C. Porter

**Affiliations:** ^1^Department of Respiratory Therapy, Faculty of Medical Rehabilitation Sciences, King Abdulaziz University, Jeddah, Saudi Arabia; ^2^Respiratory Therapy Unit, King Abdulaziz University Hospital, Jeddah, Saudi Arabia; ^3^School of Health Sciences, University of Birmingham, Edgbaston, United Kingdom; ^4^Birmingham Regional Interstitial Lung Disease Service, The Birmingham Chest Clinic, University Hospitals Birmingham NHS Trust, Birmingham, United Kingdom; ^5^UCL Respiratory, University College London, London, United Kingdom; ^6^Satsuma Lab, Centre for Medical Image Computing, University College London Respiratory, London, United Kingdom; ^7^Institute of Psychiatry, Psychology and Neuroscience, King’s College London, London, United Kingdom; ^8^Pulmonary and Critical Care Department, University of Toledo, Toledo, OH, United States; ^9^NIHR Biomedical Research Centre at South London and Maudsley NHS Foundation Trust and King’s College London, London, United Kingdom; ^10^Institute of Health Informatics, University College London, London, United Kingdom; ^11^NIHR Biomedical Research Centre at University College London Hospitals, NHS Foundation Trust, London, United Kingdom

**Keywords:** telehealth, digital health, mobile health (mHealth), interstitial lung diease, chronic obstructive pulmonary disease, respiratory disease, artificial intelligence, spirometry

## Abstract

This review provides a detailed overview of how digital health can be utilized in the management of Interstitial Lung Disease (ILD), and Chronic Obstructive Pulmonary Disease (COPD). ILD encompasses a diverse range of lung disorders characterized by inflammation and scarring of lung tissue, leading to restrictive lung physiology and impaired gas exchange, with symptoms including progressive dyspnoea, cough, and hypoxia. COPD which ranks as the third leading cause of death globally, is characterized by chronic lung inflammation causing irreversible airflow obstruction, recurrent exacerbations. While recent advances in digital health have shown promise, predicting disease progression in patients with ILD and exacerbation in patients with COPD remains challenging. This review explores the role of digital health in managing ILD and COPD, particularly focusing on telehealth and digital health technologies. Telehealth, defined broadly as the use of electronic information and telecommunications technologies in healthcare, has become increasingly relevant, especially during the COVID-19 pandemic. This review examines the role of digital health technologies in the management of ILD and COPD, with particular focus on telemedicine, and digital health tools. Remote monitoring technologies, including home spirometry and wearable devices, have demonstrated feasibility in managing respiratory diseases. However, challenges such as evidence, data reliability, varying adherence, education, and the high costs of data collection and lack of qualified clinicians present barriers for many national health systems.

## Introduction

Telehealth is increasingly recognized as a transformative approach in chronic disease management, particularly for respiratory conditions, with the potential to enhance patient monitoring and inform clinical decision-making (1). By enabling digital health and data collection, digital health tools can potentially improve self-management and deliver timely clinical insights (1). The COVID-19 pandemic has not only driven the adoption of digital health solutions, but has also played a significant role in shaping the World Health Organisation’s global strategy for digital health 2020–2025 (1). Providing access to remote health care services remotely can aid case prioritization and timely intervention, therefore reducing the increased load on health care system from direct patients visits thus reducing the costs of hospitalization, transportation, and exposure to hospital infectious disease (2–5). To fully realize the potential of digital health of physiological parameters and symptoms, an emphasis must be placed on producing high-quality data and evidence. However, digital health can only be the adopted after assessing the feasibility, utility/usage, and acceptability to patients with lung diseases.

This review provides an updated, in-depth overview of how digital health technologies are used to monitor and manage chronic respiratory conditions, particularly interstitial lung disease (ILD) and chronic obstructive pulmonary disease (COPD). By synthesizing the most recent evidence, we demonstrate the benefits, challenges, and future directions for digital health in respiratory medicine.

## Method

A comprehensive search of PubMed, Ovid-MEDLINE, and Google Scholar was conducted to identify relevant studies published prior to November 2023. References were reviewed for additional articles. MeSH terms were organized into categories related to diseases (e.g., “interstitial lung disease,” “idiopathic pulmonary fibrosis,” “COPD”), technologies (e.g., “telehealth,” “remote patient monitoring,” “wearables,” “home spirometry,” “mobile health,” “mHealth,” “Internet of Medical Things”), and outcomes (e.g., “exacerbations,” “disease progression,” “symptom monitoring,” “adherence,” “cost-effectiveness”). Eligible studies investigated digital health solutions in chronic respiratory disease care, provided data on outcomes including feasibility, usability, adherence, or clinical efficacy, and published in peer-reviewed journals in English. Initial search results were screened for relevance, followed by full text review of eligible studies. Data were extracted using a standardized template capturing study details, intervention type, and key outcomes.

The findings were synthesized into themes:

1. Global Burden of Disease: Epidemiology, economic costs, and care gaps in underserved areas.
2. Telehealth and RPM: Applications in exacerbation detection, patient engagement, and adaptations following COVID-19.
3. IoMT Infrastructure: Use of wearable sensors, AI-driven analytics, and data transmission challenges.
4. Clinical Applications: Feasibility and reliability of home spirometry, ePROM integration, and early detection of disease progression.
5. Patient Engagement: Adherence rates, psychological impacts, and barriers such as digital literacy.
6. Implementation Barriers: Usability challenges, cost-effectiveness, regulatory hurdles, and interoperability issues.
7. Future Directions: Standardization, AI/ML integration, and equity-focused policies to improve access and sustainability.

Given the narrative design of this review, formal quality appraisal tools like the RoB 2 or Newcastle-Ottawa Scale were not applied. The study also did not follow a pre-defined registered protocol or systematic review standards such as those outlined in the PRISMA guidelines.

### Global burden of respiratory diseases

Respiratory diseases encompass a wide range of different conditions that affect all individuals across all ages, presenting different symptoms and prognoses (6). The global burden of respiratory disease continues to rise, with high incidence and mortality rates becoming a serious concern (7–9). In 2017, approximately 544.9 million people worldwide were affected by chronic respiratory diseases (7, 8, 10). Given the diverse progression patterns of these diseases, predicting their actual costs and long-term outcomes remains a challenge (6). In the UK, Asthma + Lung UK estimates that 12.7 million people live with respiratory disease, including 1.2 million diagnosed with chronic obstructive pulmonary disease (COPD) (11), a condition that ranks as the third leading cause of death globally (10), and 150,000 were diagnosed with Interstitial Lung Disease (ILD) (11, 12). A recent Asthma + Lung UK report, indicates that respiratory diseases cost the UK £11 billion annually, with 29% of that expenditure allocated to COPD (7, 13). New research demonstrated the importance of early identification of exacerbations in COPD (14, 15); thus, continuous monitoring of symptoms and physiological parameters have the potential to allow earlier intervention (15). There remains a gap in the care of patients with respiratory conditions (13, 16). Particularly those with chronic respiratory diseases and those recently discharged from hospital, where changes in patients’ health are not well predicted. In the face of this growing global burden, emerging telehealth solutions offer possibilities for improved patient monitoring and resources allocation, as we discuss in the following section.

Recent studies demonstrated the significance of timely identification of worsening respiratory symptoms (14, 15, 17–19); therefore, longitudinal monitoring of physiological parameters and symptoms have the potential to allow earlier identification (20). There is an unmet need in the care of patients with respiratory diseases (13, 16). Chronic respiratory diseases are especially at risk, with limited knowledge about changes in their symptoms and physiological parameters (21–23). Emerging modalities for remote data collection, such as home based spirometry, pulse oximeters, wearables and smartphone apps may offer potential to enhance self-management and offer better, timely information for clinical assessment (18). Digital health could serve as link between hospital care and home care for these patients (24, 25). Nevertheless, uncertainties persist regarding the feasibility and acceptability of digital health in monitoring symptoms and physiology for patients with respiratory diseases.

### Telehealth and remote patient monitoring

#### Definitions and scope

Telehealth is a broad term that was defined previously as “the use of electronic information and telecommunications technologies to support and promote long-distance clinical health care, patient and professional health-related education, public health, and health administration, where technologies include video conferencing, the internet, store-and-forward imaging, streaming media, and terrestrial and wireless communications” (26). In particular, the Health Resources and Services Administration (HRSA) of the U.S. Department of Health and Human Services defines remote patient monitoring (RPM) as “the use of connected electronic tools to record personal health and medical data in one location for review by a provider in another location, usually at a different time” (26). In the United Kingdom, and according to the New England Journal of Medicine, telehealth is “the delivery and facilitation of health and health-related services including medical care, provider and patient education, health information services, and self-care via telecommunications and digital communication technologies (27). Live video conferencing, mobile health apps, ‘store and forward’ electronic transmission, and remote patient monitoring (RPM) are examples of technologies used in telehealth,” (27). Remote patient monitoring was defined as “the reporting, collection, transmission, and evaluation of patient health data through electronic devices such as wearables, mobile devices, smartphone apps, and internet-enabled computers. RPM technologies remind patients to weigh themselves and transmit the measurements to their physicians (27). Wearables and other electronic monitoring devices are being used to collect and transfer vital sign data including blood pressures, cardiac stats, oxygen levels, and respiratory rates” (27). The healthcare industry is going through rapid transformation, and new developments such as the Internet of Things and artificial intelligence are expected to be a driving force in this transformation. While telehealth and PRM improve patient access to health care, integrating these approaches with Internet of Medical Things (IoMT) technologies can further transform the management of chronic conditions, as we explore next.

### The role of the internet of medical things

**Figure 1:**
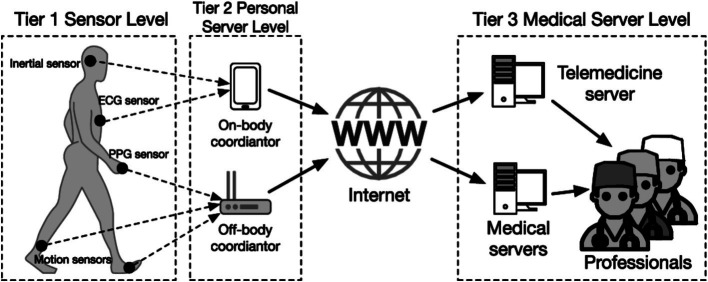
Architecture of IoMT-based healthcare system. The diagram illustrates a three-tier telemedicine system: Tier 1 with sensors—ECG (Electrocardiogram) for heart activity, PPG (Photoplethysmogram) for heart rate, and inertial and motion sensors.

The Internet of Things (IoT), first mentioned by Ashton (28), was defined as “a global infrastructure for the information society, enabling advanced services by interconnecting (physical and virtual) things based on existing and evolving interoperable information and communication technologies according to the Internet of Things Global Standards Initiative” (Figure 1) (29). Artificial intelligence (AI), on the other hand, is defined as “the capability of a machine to imitate intelligent human behavior” (30).

### The internet of medical things

**Figure 2:**
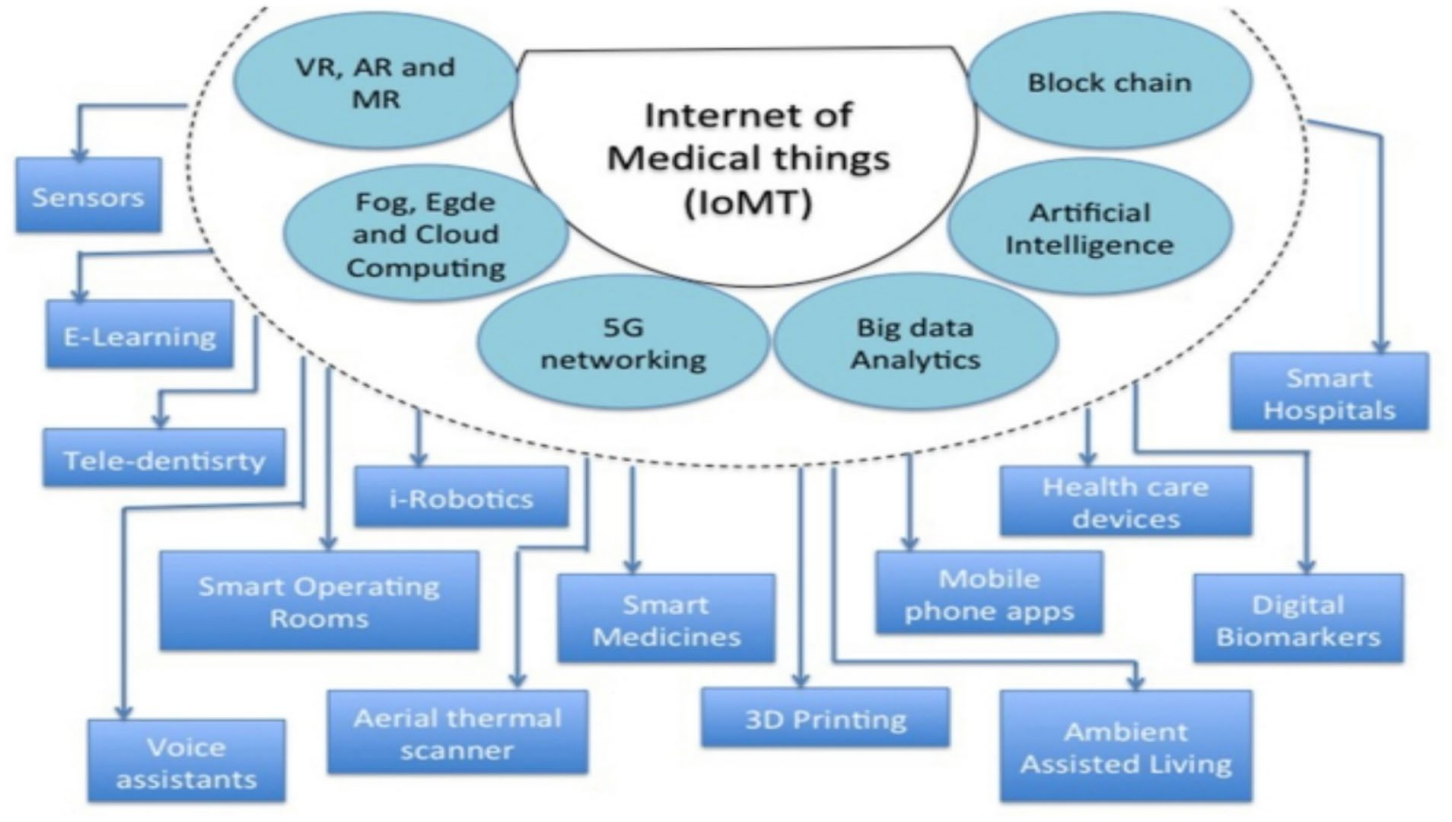
IoMT (Internet of Medical Things): A central system integrating health technologies for efficient patient care and data management. The centralized system connecting diverse health technologies to streamline patient treatment, data management, and medical services (91).

The number of IoT devices has grown exponentially since 1999 (31). There are now an estimated 10 billion connected IoT devices, and this number is expected to reach 25 billion by 2025 (31, 32). IoT technology is already being used in healthcare applications, such as remote patient monitoring, medication adherence tracking, and surgical robotics. As IoT technology continues to develop, we can expect to see even more innovative healthcare applications emerge. The global market for IoT technology is expected to reach $6.2 trillion by 2025, with 30% of that coming from healthcare (33). This means that the healthcare industry will be a major driver of IoT growth in the coming years. In addition, the Internet of Medical Things (IoMT), sometimes referred to as the Internet of Healthcare Things (IoHT), is improving healthcare (Figure 2). In particular, remote patient monitoring via wearables (34), smartphone applications, and Bluetooth devices has increased dramatically (35). These IoMT driven innovations are already influencing the management of chronic respiratory diseases, particularly COPD and ILD, which will be the focus of the following sections.

### Applications in chronic respiratory diseases

Telehealth and remote monitoring are utilized globally for managing chronic respiratory diseases and in the United Kingdom, particularly through the National Health Service (NHS). The NHS has integrated a range of technology-enabled care services (TECS) to support patients with long-term conditions. These services include telehealth, telecare, telemedicine, telecoaching, and self-care apps. These technologies empower patients to manage their healthcare more independently and efficiently from home, improving access and reducing the need for in-person visits (1, 2). During the COVID-19 pandemic, the NHS significantly expanded its digital health capabilities. For example, over 487,000 people were supported at home with digital health and remote monitoring technologies through national funding between November 2020 and January 2023. These initiatives have been critical for managing chronic conditions such as Chronic Obstructive Pulmonary Disease (COPD) and heart failure, as well as for providing post-operative care and monitoring for mental health conditions (2). In North America, particularly in the United States and Canada, telehealth has become widely adopted for remote consultations, virtual care, and remote monitoring services. European countries like the United Kingdom, Norway, Sweden, Denmark, Germany, and Italy are at the forefront of telehealth adoption, utilizing it to reach patients in remote areas and enhance access to care. In Asia, countries such as China, Japan, and South Korea have embraced telehealth to address healthcare access challenges, especially in rural areas. Additionally, Australia leads telehealth utilization in the Australasian region. Despite infrastructural challenges, countries like South Africa and Kenya in Africa, as well as Brazil, Argentina, and Chile in South America, have implemented successful telehealth initiatives to improve healthcare access in underserved regions. In the Middle East, several countries have made significant progress in their utilization of telehealth services, integrating it extensively into their healthcare systems (36).

The advent of daily remote monitoring, particularly home based spirometry, offers a non-invasive solution to several challenges faced by ILD patients (19, 37, 38). These challenges include the infrequency of spirometry monitoring every 3 months at clinics, the high costs associated with hospital visits, and the inconvenience of commuting for tests (18). Additionally, in-clinic spirometry often faces delays due to high demand, with wait times exceeding 3months in some cases (39). There is also a risk of infection exposure during clinic visits, which has become particularly concerning during the COVID-19 pandemic (40). Digital health facilitates continuous monitoring of disease, and timely adjustments to treatment plans, which can significantly enhance patient management and outcomes (19). Furthermore, it can enhance the accuracy of treatment effect estimates by enabling more frequent measurements, improve patient engagement by encouraging active participation in their own care, and reduce the burden on healthcare systems by minimizing the need for frequent clinic visits (19, 41–43). In addition, remote patient monitoring, involving more than just FVC, may provide a more comprehensive and real-time assessment of physiological parameters and symptoms, addressing the limitations of conventional indicators. These devices offer a reliable way to identify patients at higher risk for developing AE-ILD, improve understanding of AE-PF clinical progression, and may facilitate the early detection of AE-IPF (19). With evidence supporting the ability of home handheld spirometry to improve clinical trail outcomes in IPF therapeutics, this approach can be highly valuable (18, 19), attention should be directed toward studies investigating the advantages of daily home handheld spirometry for patients with ILD. This naturally extends into the wider application of digital health. By utilizing questionnaire responses to monitor symptoms like fatigue, cough, anxiety, depression, and QoL, as well as monitoring key physiological parameters such as respiratory rate, oxygen saturation, heart rate, and FVC, a holistic understanding of the patient’s condition can be obtained, promoting an effective disease management strategy.

In addition, mobile health (mHealth) refers to “health care applications and programs patients use on their smartphones, tablets, or laptops. These applications allow patients to track health measurements, set medication and appointment reminders, and share information with clinicians” (27). According to the World Health Organisation (WHO), mobile health, also known as mHealth, is defined as a term used for medical and public health practice supported by mobile devices, such as mobile phones, patient monitoring devices, personal digital assistants (PDAs), and other wireless devices (44). “mHealth applications include the use of mobile devices in collecting community and clinical health data, delivery of health care information to practitioners, researchers, and patients, real-time monitoring of patient vital signs, and direct provision of care” (44). Telehealth was also defined as remote monitoring of patients in their homes to predict exacerbations early and build their self-care competencies, according to the NHS Commissioning Assembly (45). Finally, the health care industry is going through rapid transformation, and new developments such as the Internet of Things and artificial intelligence are expected to be a driving force in this transformation.

### Applications in COPD management

The World Health Organisation reported that COPD is the third leading cause of death worldwide (10). In 2019, it was estimated to have caused 3.23 million deaths globally (10). COPD is characterized by inflammation of the lung, which causes irreversible airflow obstruction and, generally, an accelerated decline in lung function (6). Patients with COPD are prone to recurrent chest infections known as acute exacerbations (6, 46). Acute exacerbation of chronic obstructive pulmonary disease (AECOPD) is defined as an acute change of respiratory symptoms needing a change in treatment, and these events are generally associated with deterioration of lung function and QoL (46, 47). Previous studies suggest that longitudinal monitoring of physiological parameters and symptoms have the potential to provide earlier detection of exacerbation, but a history of frequent exacerbations and patient reporting remains the gold standard of exacerbation prediction and identification (48). Recent studies demonstrated the significance of timely identification of the exacerbation of COPD (6). Thus, patients with COPD could be continuously monitored outside clinical settings to mitigate the impact of exacerbation on health status (48).

Remote monitoring for exacerbation of COPD may be a valuable approach to facilitate appropriate health care. Remote monitoring of physiological parameters (respiratory rate, heart rate, and oxygen saturation) and self-reported outcomes both offer a unique combination that allows for earlier detection of AECOPD. This could aid in timely intervention, minimize exacerbation severity, and prevent hospitalization.

The findings from recent studies highlight the promising role of telehealth interventions in managing COPD (49). Telemonitoring, in particular, has demonstrated efficacy in reducing the frequency of emergency room visits, although its impact on hospitalization rates remains uncertain (50). High levels of patient satisfaction indicate that telemonitoring is well-accepted and can be effectively integrated into COPD care plans (51). Moreover, telemedicine interventions have been associated with reductions in respiratory exacerbations and improvements in physical activity levels and quality of life, suggesting their potential to supplement standard COPD care (52, 53). Despite these benefits, the variability in effectiveness across studies underscores the need for further high-quality research to establish standardized protocols and best practices (54). Wearable technology interventions also show promise, particularly in increasing physical activity, though their impact on quality-of-life measures and exacerbation prediction requires further investigation (49). Overall, these studies suggest that integrating telehealth into COPD management strategies can enhance clinical outcomes and patient experiences while potentially reducing the burden on healthcare systems. Building on these approaches in COPD, digital health interventions have also shown promise in detecting progression and managing patients with interstitial lung disease, discussed in the next section. A summary table of the included studies on digital health interventions in ILD is shown in Table 1.

**Table 1:** Summary of included studies on digital health interventions in ILD.

Author (year)	Country	Study design	Sample size	Disease group	Measures/frequency	Study length	Key outcomes
Moor et al. (41)	Netherlands	RCT	*n* = 90	IPF	FVC (daily), K-BILD, PESaM, EQ-5D-5L, HADS, VAS	24 weeks	Improved psychological well-being, high correlation home vs. hospital spirometry
Maher et al. (70)	United States	RCT	*n* = 253	Unclassifiable ILD	FVC (daily), 6MWD, UCSD-SOBQ, SGRQ	24 weeks	Variability in home spirometry, FVC decline less in pirfenidone group
Russell et al. (58)	UK	PCS	*n* = 50	IPF	FEV1, FVC (daily)	279 days	Daily FVC predictive of disease progression and mortality
Johannson et al. (64)	United States	PCS	*n* = 25	IPF	FEV1, FVC (3x weekly), UCSD-SOBQ, Dyspnoea-VAS	24 weeks	High adherence to home spirometry, reliable FVC and dyspnoea tracking
Veit et al. (69)	Germany	PCS	*n* = 47	ILD	FVC (3x daily), 6MWD, DLCO, SGRQ, VAS	6 months	Adherence higher in first 3 months, strong correlation home vs. hospital spirometry
Edwards et al. (68)	Ireland/United States	PCS	*n* = 36	PF	FVC (daily), mMRC (daily), IPF-PROM (weekly)	1 year	Positive impact on well-being, good correlation home vs. hospital spirometry
Moor et al. (65)	Netherlands	PCS	*n* = 10	Sarcoidosis	PEF, FEV1, FVC (daily), PROM, KSQ, EQ-5D-5L	4 weeks	High correlation between home and hospital FVC, 94.6% adherence
Moor et al. (43)	Netherlands	PCS	*n* = 10	IPF	Home spirometry (daily), K-BILD, HADS, EQ-5D-5L	4 weeks	Home spirometry correlated with hospital results, 98.8% adherence
Moor et al. (59)	Netherlands	PCS	*n* = 50	IPF	FVC (2x daily), K-BILD	6 weeks	Morning FVC higher than afternoon, step count correlated with FVC
Broos et al. (60)	Netherlands	PCS	*n* = 21	Sarcoidosis	FVC (daily), MRC, FAS, SGRQ, SF-36	3 months	Reliable home spirometry for sarcoidosis
Marcoux et al. (67)	Netherlands	PCS	*n* = 20	IPF	FVC (3 maneuvers daily), 6MWD	12 weeks	Strong correlation between office-based and handheld FVC
Noth et al. (92)	Netherlands	PCS	*n* = 346	IPF	FVC (weekly)	1 year	Strong home vs. clinic FVC correlation but weaker rate of decline
Moor et al. (59)	Netherlands	PCS	*n* = 10	Systemic sclerosis associated ILD	FVC (daily), K-BILD, HADS, EQ-5D-5L	3 months	High adherence (98.8%), 90% found home monitoring pleasant

### Application in ILD management

#### Home handheld spirometry

Home handheld spirometry has gained increased attention, especially in recent times in light of the COVID-19 pandemic lockdown. Indeed, home handheld spirometry linked to smartphone apps have emerged as a contemporary solution to reducing the delays and risk of infection associated with hospitals (38). A newly developed devices for smart handheld spirometry facilitates the continuous measurement of FVC at home (18, 38, 55–57). This device would need to be connected via Bluetooth to a smartphone app, which means that patients are able to see their results in real-time through their smartphone. Moreover, clinicians can monitor the patient’s spirometry using a web portal developed specifically for this task (43, 58–62). Numerous studies over the last decade investigated the use of a home handheld spirometry in patients with interstitial lung disease (ILD) (18, 38, 55–57). In general, this approach is feasible, reliable, acceptable, user-friendly and it results in high participant satisfaction (18, 63). However, several cautions and drawbacks associated with home handheld spirometry have been also reported (38). This section summarizes existing literature on home handheld spirometry.

In 2016, Russell et al. (58) conducted a single-center prospective study with the aim of assessing the feasibility and reliability of home handheld spirometry. Therein, patients with idiopathic pulmonary fibrosis (IPF) were requested to conduct daily FVC and forced expiratory volume (FEV1) measurements for up to 490 days. The 50 patients who took part in the study recorded for a median of 279 days (range 13–490 days) (58). Although no adherence percentage was reported, the results suggested that daily home handheld spirometry is feasible. Indeed, the study analyzed the FVC to determine the correlation between hospital and home measurements, and findings showed a strong correlation (58). The rate of FVC decline showed an association with mortality during the first 3 months, with high statistical significance (*p*-value < 0.0001) (58). However, the study found that FVC readings taken at home were typically lower compared with hospital readings. In addition, in some cases, home handheld discontinued because of cough, absence of flow volume or concerns about measurement quality. Finally, the need for patients to keep a diary of daily spirometry measurements raised concerns about data reliability (58).

In another study, Johannson et al. (64) conducted a single-center prospective study over 40 weeks investigating both the feasibility and reliability of a home handheld spirometer. In this study, 25 patients with IPF were requested to conduct weekly measurements of FVC using a personalized handheld spirometer. Although a decline in patient adherence was reported over time, adherence was generally good (90.5%), and a strong correlation between home based and hospital FVC measurements was observed (*r* = 0.91). However, 13% of patients found the spirometer challenging to use (64).

In a national multi-center prospective observational study conducted by Broos et al. (60), the effect of early steroid treatment on FVC was investigated. The study involved daily monitoring of 21 patients diagnosed with sarcoidosis over a study period of 3 months. Findings suggest that using a daily home handheld spirometry can potentially serve as a useful tool in monitoring steroid treatment effects in patients with sarcoidosis. However, the study lacked data on patient adherence to home handheld spirometry, which is a critical factor in assessing the success of this monitoring method. Furthermore, the study used specific software (Micro Diary; Carefusion), which was operated by the researchers for downloading readings, and the functionality of which may have had an influence on the data recording process (60).

Catharina et al. (65) carried out a single-center prospective study on 10 patients with IPF over 4 weeks to assess the feasibility of utilizing a wireless home handheld spirometry, using the MIR Spirobank smart spirometer. Results showed a high correlation between home and hospital spirometry measurements for both FVC and FEV1 (*r* = 0.94, *p* < 0.001 and *r* = 0.97, *p* < 0.001 respectively), and a mean patient adherence of 94.6%. This pilot study was followed by another single-center prospective study in 2019 involving 10 patients with sarcoidosis over a four-week study period (42). Again, high correlations were observed between home and hospital FVC and FEV1 measurements (*r* = 0.97, *p* < 0.001 and *r* = 0.96, *p* < 0.001), and a mean patient adherence of 94.6% (42).

Moor et al. (59) conducted a further prospective observational study involving 50 patients with fibrotic ILD. In this study, FVC was measured twice daily over 6 weeks to evaluate its diurnal variation. Results indicated FVC is higher in the morning than in the afternoon, but several technical issues imply data that may be missing. In another randomized controlled study comparing home monitoring with standard care in 90 IPF patients (66), home monitoring appeared to be feasible and reliable, with home and hospital measurements being strongly correlated (*r* = 0.97 at baseline and 12 weeks, *r* = 0.96 at 24 weeks) with a mean adherence of 93%, this allowed for tailored medication adjustment and enhanced psychological well-being relative to standard care alone (mean difference 1.04 points; 95% CI, 0.09–2.00; *p* = 0.032) (66).

Another single-center prospective study by Moor et al. (56) involved the observation of 10 patients with systemic sclerosis-ILD over 3 months to determine the feasibility and optimal frequency of online home handheld spirometry. The authors found that readings from home handheld spirometry readings were highly correlated with readings from hospital spirometers (*r* = 0.99, *p* < 0.001). The mean adherence to home handheld spirometry herein was 98.8% (SD 1.5) (56).

Another single-center observational study conducted by Marcoux et al. (67) involved 20 patients with IPF over 12 weeks to assess the feasibility of daily home handheld spirometry. The results demonstrated a mean adherence of 84%, with a high correlation between home and hospital FVC values at baseline (*r* = 0.97), was shown, there was a lower correlation after 12 weeks (*r* = 0.90) (67).

A community-based participatory research program conducted by Edwards et al. (68) observed 36 patients with fibrotic ILD in two countries (24 participants from the US and 12 participants from Ireland). Herein, researchers used a mobile application called patientMpower to conduct home handheld spirometry testing for 1 year. In terms of adherence, 78% of participants recorded home handheld spirometry readings for 6 weeks, with 58% recorded home handheld spirometry for at least 180 days, and only 25% recorded home handheld spirometry for at least 360 days. The authors suggested that the independent use of the patientMpower application, without active involvement from health care providers, might have contributed to the decrease in adherence over time. Furthermore, in measuring their spirometry, 20 participants recorded oxygen saturation via Bluetooth pulse oximetry, averaging 58 times per person, and with 10 of these participants recorded oximetry for at least 180 days. The patient reported highly positive experiences with the patientMpower application, with the majority of respondents considering the application easy to use and helpful in managing their lung fibrosis. The participants also reported that the application positively affected their well-being and daily life (68).

A prospective observational study was conducted by Veit et al. (69) observing 47 patients with fibrotic ILD over 6 months to determining the prognostic potential of daily FVC measurements. Patient adherence to home handheld spirometry was 85.1%, reflecting high acceptance. Only three patients (8.5%) discontinued use within the first week; in these cases, use was discontinued because of dyspnoea. Another three (6%) were excluded because of technical issues and a poor-quality of measurements. Forty patients continued in the study for a mean duration of 161 days and performed home handheld spirometry measurements on 81.8% of those days, with 98.4% of the measurements being of good quality. Mean home FVC measurements showed a strong correlation with baseline hospital values over the first 7 days (*r* = 0.96, *p* < 0.0001) and at both the 3-month (*r* = 0.95, *p* < 0.0001) and 6-month visits (*r* = 0.93, *p* < 0.0001). Twelve patients (30%; 5 with IPF and 7 without IPF) experienced disease progression, with such outcomes as death, lung transplantation, acute exacerbation, and hospital-based FVC decline of more than 10%. Patients with progressive disease had significantly lower 6MWD values (301 ± 140 m vs. 433 ± 89 m; *p* = 0.009) and total scores on the King’s Brief Interstitial Lung Disease ILD (K-BILD) instrument (46.3 ± 8.1 vs. 55.8 ± 12.7; *p* = 0.004), indicating physical and subjective wellbeing limitations.

In the first 28 days, 60% of patients demonstrated a FVC coefficient of variation (CoV) ≥ 5, and 15% showed an FVC CoV ≥10%. The median FVC CoV was 5.9%, with a range of 3.5 to 17.8%. The progressive group showed higher variation (8.6% median FVC CoV) than the stable group did (4.8% median FVC CoV, *p* = 0.002). Over 3 months, patients with disease progression demonstrated significantly higher FVC variability (8.4 ± 3.2%) compared to stable patients (5.5 ± 2.5%; *p* = 0.002). Finally, FVC variability over 28 days was found to be independently associated with disease progression (hazard ratio 1.203; 95% CI: 1.050–1.378; *p* = 0.0076), with the optimal cut-off for distinguishing low and high variability determined to be 7.9% (69).

Maher et al. (70) conducted an international multi-center, double-blind, randomized, placebo-controlled phase 2 trial involving 253 patients with unclassifiable ILD. The study sought to assess the mean predicted change in FVC from baseline over 24 weeks using daily home handheld spirometry. However, technical difficulties and issues with data reliability prevented statistical analysis. Problems included daily FVC readings that were physiologically impossible (<0.5 L or > 6 L), with predicted increases of 33 L at 24 weeks: thus, invalidating the application of the pre-planned statistical model.

Meanwhile, Noth et al. (62) carried out an international multi-center prospective study involving 346 patients with IPF over 52 weeks to evaluate the feasibility and validity of using home handheld spirometry to measure lung function decline. While patient adherence decreased over the course of the study, the overall mean and median adherence rates remained high, at 86 and 96%, respectively. In addition, a strong correlation of home and hospital FVC was evident at all time points (*r* = 0.72–0.84). However, the variability in FVC change was higher with home handheld spirometry, and the correlation of home- and clinic-measured change in FVC was accordingly weak (*r* = 0.26) (62).

Khan et al. (71) carried out a multi-center observational cohort study over a period of 3 months, involving 82 patients with fibrotic ILD to evaluate the clinical use of home handheld spirometry as an alternative to hospital spirometry. Participants were asked to perform a single blinded, forced expiratory manoeuver on a daily basis. The full 3 months of home and hospital spirometry data were collected for only 43 participants; some patients’ hospital spirometry data were not collected consistently because of the COVID-19 pandemic, leading to the exclusion of these participants from the analysis. The median adherence to daily home handheld spirometry readings for all participants was 81%, and this increased to 91% in those who completed the 3 months study period. A good correlation between home based and hospital based spirometry was observed both at baseline (*r* = 0.89) and at 3 months (*r* = 0.82). Consistent with the high home-hospital correlation, Bland–Altman plots showed that more than 90% of home handheld spirometry values were within the agreement limits of hospital values at both time points, although home values were lower than the hospital values (71).

A study in Serbia assessed the efficiency and practicability of home handheld spirometry tests in patients with ILDs. The study found home handheld spirometry tests to have a strong correlation with office spirometry tests both at baseline and at the end of the study. Despite this, the adherence rate was only 68%. The study found that home handheld spirometry tests did not affect patients’ overall QoL or anxiety levels; indeed, patients expressed positive feedback and high satisfaction (72).

Finally, Miedema et al. (73) assessed the utility of home handheld spirometry in a multi-center prospective study wherein home handheld spirometry were used for patients with progressive asbestosis over 3 months. Using daily domiciliary spirometry readings taken over 24 weeks, the study aimed to assess the safety, efficacy and tolerability of pirfenidone in asbestosis patients with a progressive disease subtype. Prior to pirfenidone treatment, the data showed a significant decline in FVC during the 12-week observational period. Upon initiation of treatment, FVC levels remained stable throughout the 24-week treatment phase (73).

### Electronic patient-reported outcome measures and mobile health

Patient-reported outcome measures (PROMs) are self-reported measures of patient (48) that can be used to assess patient QoL and symptoms. These measures can be collected in a variety of ways, including through paper-based surveys, telephone interviews, and online questionnaires. Online collection, in particular, has several advantages. First, it is more convenient and efficient for patients, as patients can complete online surveys at their own pace. Second, online collection is more accessible, especially for patients who are unable to travel to a clinic or hospital to complete a paper-based survey, such as those who live in rural areas or have limited mobility. Third, online collection can be integrated with electronic health records, making it easier for health care providers to track patient progress and to identify those potentially in need of additional care. The validity and reliability of online PROM collection have been well-established as equal to the validity and reliability of paper collection, and its reliability may even be greater, as online collection can reduce missing data (65, 74).

In this light, electronic platforms and mobile applications have been developed to monitor patients with heart failure, cancer, and other chronic conditions; however, platforms specific for ILD are limited (18, 55, 75, 76). The uses of E-health utilities for patients with ILD suggests that such applications may be beneficial for monitoring disease trajectory and symptom management, as well as for providing support such as reminding patients to take medication and report treatment responses to clinicians. In addition, such applications can provide those with ILD accessible information and educational tools.

Recently, several studies have demonstrated the feasibility of using E-health utilities for the online collection of patient-reported outcomes (65, 68). In a recent ILD guideline, E-health was defined as “an emerging field in the intersection of medical informatics, public health and business, referring to health services and information delivered or enhanced through the Internet and related technologies” (77). Catharina et al. (65) had patients use the home monitoring program IPF-online to submit four weekly reports of side effects and symptoms. This was followed by another study in which patients with sarcoidosis used the web application Sarconline to report VAS fatigue, dyspnoea, cough, and well-being each week. The overall patient experience was positive, with an overwhelming majority (90%) finding the Sarconline application to be user-friendly, and the daily spirometry, activity tracking, and PROM were reported to not be burdensome. Finally, the same group conducted a randomized control trial in which patients with IPF performed daily home handheld spirometry and used IPF-online for weekly reports of K-BILD, PESaM, EQ-5D-5L, HADS, VAS, GRC, and EQ-VAS. Patient satisfaction with and use of the home-monitoring program were notably high; the median adherence to daily home handheld spirometry was 97%, and the PROM completion rate was 93%. The patients were generally appreciative of the home monitoring program, with 95% stating that they would recommend it to others. A significant number reported that the program gave them insight into their disease course, made them feel reassured, and that the program facilitated easier communication with the hospital. In addition, the use of home-monitoring resulted in improvement of the mean total K-BILD score over the 24-week study period (2.70 ± 9.5 points, as compared to a negligible 0.03 ± 10.4 with standard care). However, the between-group difference of 2.67 points did not reach statistical significance (95% CI, −1.85 to 7.17; *i* = 0.24). The K-BILD psychological domain showed an increase of 5.12 (±15.8) points in the home monitoring group and a slight decline of 0.48 (±13.3) points in the standard care group; the between-group difference of 5.6 points (95% CI, −1.13 to 12.3; *p* = 0.10) indicated a positive yet statistically insignificant trend in psychological well-being for home-monitored patients. With regard to medication use and hospital visits, the home monitoring group showed more frequent adjustment of medication during the study, primarily due to side effects (41–43, 56, 59, 65, 66, 78).

Separately, Edward et al. (68) evaluated a newly developed mobile application called patientMpower, which was designed specifically to assess IPF symptoms and their impact on patient life via the IPF patient-reported outcome measure (IPF-PROM). In addition, patientMpower facilitates the use of home handheld spirometry. In this study, modified medical research council (mMRC) was collected once daily and IPF-PROM weekly. The results showed home use of patientMpower to be feasible and acceptable for patients with IPF (68).

Taken together, these findings indicate that developing a tool for the continual assessment of patient-reported outcomes is critical to accurately diagnosing and managing ILD (19, 79, 80). The validated tools used to collect patient-reported outcomes focus on emotional symptoms, response to therapy, well-being, and health-related QoL. The current gold standard endpoint measurement in clinical trials for idiopathic pulmonary fibrosis (IPF) is forced vital capacity (FVC), which is used for assessing disease progression and treatment efficacy (81). Nonetheless, physical activity, symptom burden, and HRQoL all convey important information; and given the association of subjective and objective measures, both data types may contribute to predicting significant events and disease trajectory. Recently, an international interdisciplinary expert reported the top patient-centered outcome in patients with ILD. The patient-centered outcomes focus on the issues of greatest concern to patients, includes; (1) health related QoL (general and disease-specific) (2) functional ability (daily living activities, ability to function at home and at work, ability to leave home for leisure or travel) (3) emotional and psychological well-being (anxiety, depression, freedom, grief, stress, emotional confidence) (4) symptoms (dyspnoea, cough, fatigue, medication side effects) (5) knowledge acquisition (therapeutic options and ongoing clinical trials, advantages and disadvantages of tests and interventions, prognosis and level of certainty for what the future holds, and the supportive care options) (6) hospitalizations and survival (hospital-free days) (7) assessing the need for oxygen (the prescribed dosage, the type and availability devices for oxygen delivery, along with cost and logistical concerns) (80, 82). Although home handheld spirometry and electronic PROMs have produced encouraging results, broader concerns around device usability, data quality, and long term adherence persist, as the next section examines.

### Wearables and physical activity trackers

Wearables are defined as electronic devices that can be worn by individuals on the body to track physiological parameters such as respiratory rate, heart rate, physical activity, and sleep patterns (83). These devices include wristbands, trackers, smartwatches, and clothing with sensors. The collected data are transmitted wirelessly to a smartphone app or computer for continued tracking.

The WHO has defined physical activity as “any bodily movement produced by skeletal muscles that requires energy expenditure—including activities undertaken while working, playing, carrying out household chores, traveling, and engaging in recreational pursuits” (84). Exercise capacity refer to an individual’s ability to endure physical activity, often involving different exercises to boost physical health.

As physical activity is known to be associated with symptoms, physical activity trackers are widely used in research involving patients with ILD (85). Root et al. (85) were among the first to report the use of a physical activity tracker in patients with idiopathic pulmonary fibrosis (85). Patients wore accelerometers and GPS trackers for seven consecutive days. Despite the short duration of the study, “Steps per day were correlated with symptoms and several quality-of-life domains.” Bahmer et al. (86) found a significant decline in daily physical activity in patients with IPF, and this decline was strongly associated with factors such as increased fatigue, impaired lung function, and reduced exercise capacity (86).

Several studies have used the Fitbit Flex 2 device (Fitbit, Inc., San Francisco, CA, United States), in patients with ILD, specifically to record daily steps taken, distance walked, and active minutes (42, 67). Moor et al. reported patients with sarcoidosis using the Fitbit Flex 2 had a daily mean adherence of 91.3% (42). In a prospective cohort study of patients with IPF by Marcoux et al. (67), patients wore the Flex 2 during their waking hours, and the data uploaded to the Fitbit portal were made accessible to both study personnel and patients due to the study’s exploratory nature. With a mean adherence of 91%, this study demonstrated that wearing a physical activity tracker is feasible over a period of 12 weeks (67). Despite these technological advances, implementing wearables and other digital solutions on a larger scale remains challenging, as we discuss in the following section on future directions.

### Challenges and future directions

While this comprehensive review provides valuable insights into digital health for respiratory diseases, several challenges currently limit the utility of digital health for managing these conditions (18, 39, 49, 55, 87, 88). Usability remains a primary concern, especially for older adults and patients with limited digital literacy, who may benefit from more robust educational support and simpler device interfaces (55, 87, 89). In addition, the lack of clinical validation for consumer-grade wearable data reduces trust and poses challenges for clinicians interpreting this information outside of controlled settings (88). Moreover, the high costs of purchasing, maintaining, and integrating these technologies create sustainability issues for national health systems, especially in rural or lower-income areas with limited access to digital devices and internet infrastructure (39, 55, 87–89). To address the cost and resource demands, including the recruitment of skilled staff for data management, scalable solutions and advanced data integration, such as automatic data sharing and remote training are important (55, 90).

**Figure 3:**
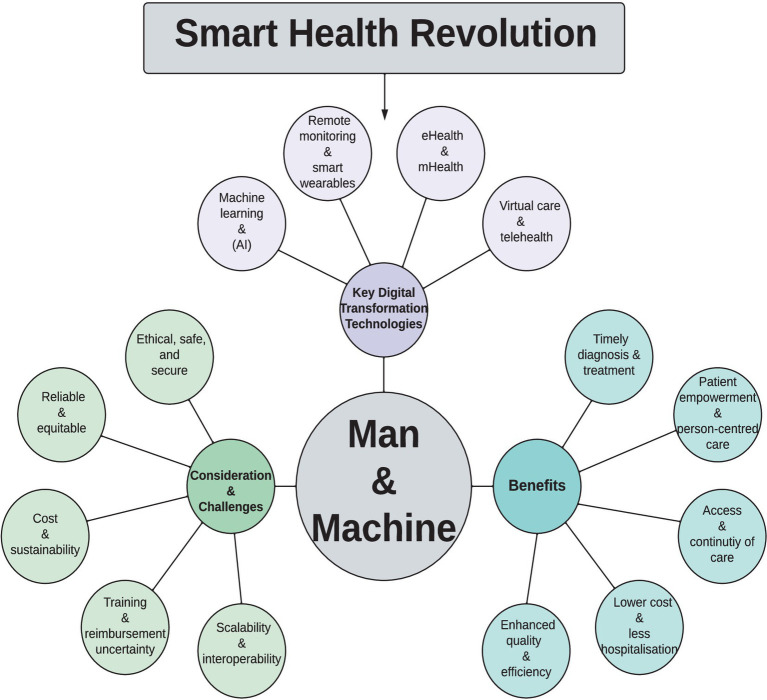
Smart health revolution. Overview of key digital transformation technologies in healthcare: benefits, considerations, and challenges.

Data privacy and security concerns further impact patient engagement, also the potential psychological effects of continuous monitoring, and the burden of manual self-reporting which may reduce adherence over time (39, 87–89). Less intrusive, passive devices could potentially improve long-term comfort and engagement (55). Manual entry may demand more patient engagement and introduce variability, whereas automated collection can reduce user burden but requires thorough validation to ensure accuracy and data integrity. Finally, variability in healthcare infrastructure and funding across regions highlights the need for further research to assess digital health’s cost-effectiveness and universal applicability across diverse healthcare settings (19) (Figure 3). Figure 3 illustrates the concept of a “Smart Health Revolution” by presenting key digital transformation technologies in healthcare, including remote monitoring, smart wearables, eHealth, mHealth, telehealth, virtual care, machine learning, and Artificial Intelligence. This figure highlights the benefits of these technologies, such as timely treatment and patient empowerment, while also addressing challenges related to cost, security, and scalability. In addition, many devices still lack standardized protocols for data integrity and calibration, emphasizing the need for robust clinical trials, including large-scale randomized studies to validate both cost-effectiveness and real-world utility. Clearer regulatory frameworks and reimbursement models must also be established to ensure data security, equitable access, and sustainable implementation. Collaboration among researchers, clinicians, policymakers, and technology developers will be critical to advance these efforts, thereby bridging the gap between innovation and meaningful clinical practice.

## Conclusion

In summary, digital health has proved to be a feasible, contemporary approach to healthcare, as it has bridged the gap between hospital and home for these patients. In particular, remote monitoring after hospital admission may empower patietns, improve access to care, and enable earlier detection of clinical deterioration. However, transitioning home monitoring from a research setting to standard practice will require robust evidence of its efficacy, cost effectiveness, and acceptability. Larger scale randomized controlled trails are needed to clarify the benefits of digital health interventions for ILD and other respiratory conditions, thereby informing the development of licnical guidelines and protocols. In addition, comparative effectiveness research could help to transition home monitoring from research setting to standard care, robust evidence of its efficacy, cost-effectiveness, and acceptability is essential. Large-scale randomized controlled trials (RCTs) are needed to provide conclusive data on its benefits for ILD patients, establishing clinical guidelines and protocols for routine integration. Comparative effectiveness research could further clarify the advantages of home monitoring over traditional methods, ultimately facilitating widespread adoption and improving patient outcomes.
